# Knowledge of modifiable risk factors of heart disease among patients with acute myocardial infarction in Karachi, Pakistan: a cross sectional study

**DOI:** 10.1186/1471-2261-6-18

**Published:** 2006-04-27

**Authors:** Muhammad S Khan, Fahim H Jafary, Tazeen H Jafar, Azhar M Faruqui, Syed I Rasool, Juanita Hatcher, Nish Chaturvedi

**Affiliations:** 1 Clinical Epidemiology Unit, Department of Community Health Sciences, Aga Khan University, Karachi, Pakistan; 2 Section of Cardiology, Departments of Medicine, Aga Khan University, Karachi, Pakistan; 3 National Institute of Cardiovascular Diseases, Karachi, Pakistan; 4 National Heart and Lung Institute, Imperial College London, UK

## Abstract

**Background:**

Knowledge is an important pre-requisite for implementing both primary as well as secondary preventive strategies for cardiovascular disease (CVD). There are no estimates of the level of knowledge of risk factor of heart disease in patients with CVD. We estimated the level of knowledge of modifiable risk factors and determined the factors associated with good level of knowledge among patients presenting with their first acute myocardial infarction (AMI) in a tertiary care hospital in Karachi, Pakistan.

**Methods:**

A hospital based cross-sectional study was conducted at the National Institute of Cardiovascular Disease, a major tertiary care hospital in Karachi Pakistan. Patients admitted with their first AMI were eligible to participate. Standard questionnaire was used to interview 720 subjects. Knowledge of four modifiable risk factors of heart disease: fatty food consumption, smoking, obesity and exercise were assessed. The participants knowing three out of four risk factors were regarded as having a good level of knowledge. A multiple logistic regression model was constructed to identify the determinants of good level of knowledge.

**Results:**

The mean age (SD) was 54 (11.66) years. A mere 42% of our study population had a good level of knowledge. In multiple logistic regression analysis, independent predictors of "good" level of knowledge were (odds ratio [95% confidence interval]) more than ten years of schooling were 2.5 [1.30, 4.80] (verses no schooling at all) and nuclear family system (verses extended family system) 2.54 [1.65, 3.89]. In addition, Sindhi ethnicity OR [3.03], higher level of exercise OR [2.76] and non user of tobacco OR [2.53] were also predictors of good level of knowledge.

**Conclusion:**

Our findings highlight the lack of good level of knowledge of modifiable risk factors for heart disease among subjects admitted with AMI in Pakistan. There is urgent need for aggressive and targeted educational strategies in the Pakistani population.

## Background

Progressive urbanization, and adoption of a "western" lifestyle contributed to the rising burden of cardiovascular disease (CVD) in the developing world [1-3]. Developing nations continue to be ill-equipped to handle this burden and this coupled with poor literacy rates and lack of awareness of disease symptoms result in worse disease outcomes [4]. This is reflected in the rising rates of hospital admissions and mortality from CVD at an early age [5-7], which in turn inflate the disability adjusted life year (DALYs) [5,8]. Furthermore, People of South Asian descent have one of the highest risks of CVD in the world. Thus it is likely that escalation of the global CVD epidemic will be most marked in Pakistan and India.

Prevention of CVD is the most effective way of combating the CVD epidemic in the resource poor nations. Knowledge of modifiable risk factors (smoking, lack of exercise, obesity and consumption of fatty foods) for heart diseases has been identified as a prerequisite for change in behavior and is often targeted by prevention programs [9,10]. Although knowledge alone is insufficient, it is assumed to be a key component of behavioral change decision making [11], and provides cues for action [12]. Estimating the level of knowledge of the population at large as well as those suffering from CVD can help to guide public health programs especially those directed towards reducing modifiable risk factors for CVD. Earlier studies have revealed that education programs for the elderly were effective in improving health promotion knowledge and behaviors [13,14].

The level of knowledge of risk factors for CVD varies among different populations. In the US whites have higher level of knowledge about risk factors of CVD than others, often disadvantaged groups, such as African Americans [11]. Similarly, in the UK, South Asian families were less likely to take regular exercise, and had a lower awareness of cholesterol or dietary content (fiber, sugar, salt) compared to the native white population[15,16]. The level of education is one of the predictors of knowledge of healthy life styles. In South Asia the family is often the source of knowledge and awareness of a healthy life style. The traditional extended family household may not be as updated in current knowledge as a nuclear family household because of a more orthodox attitude to health beliefs.

A study on the risk factors for heart disease has shown that tobacco use, ghee (clarified butter) intake, raised fasting glucose, high cholesterol, paternal history of CVD, low income, and low levels of education are associated with premature myocardial infarction in Pakistan [17]. A more recent study reported very poor knowledge amongst a general population surrogate in Karachi [18]. There are no estimates of the level of knowledge in patients with CVD. The purpose of this study was to examine the level of knowledge and to determine the factors associated with a good level of knowledge of modifiable risk factors of CVD in a sample of individuals who were hospitalized with their first acute myocardial infarction (AMI). This was a part of a larger study which focused on delay in seeking early medical care after AMI onset.

## Methods

A hospital based cross-sectional study was conducted at the National Institute of Cardio-Vascular Disease (NICVD) at Karachi from July 2003 to February 2004. Although NICVD is a tertiary care government hospital, it is the initial point-of-care for the majority of patients (across the socioeconomic spectrum) with heart disease in Karachi. All subjects admitted to NICVD during the study period with their first episode of AMI and who fulfilled the AMI criteria were eligible to participate in the study. AMI was defined using the European Society of Cardiology and American College of Cardiology's criteria [19,20]. The presence of at least two of the following three factors was considered as diagnostic for AMI:

1. Typical chest pain lasting for at least 20 minutes

2. Electrocardiogram showing ST elevation of at least 2 mm in two or more contiguous leads with subsequent evolution of the ECG

3. Diagnostic cardiac marker (doubling of creatine kinase with at least 10% MB fraction) or elevated or positive troponin I (T).

The admission records of the Emergency Room of the NICVD were reviewed daily to identify patients admitted to the hospital with a first AMI. Those who survived for first 24 hours post admission were eligible for screening. A trained research medical officer approached each of these patients to determine eligibility. Patients who gave informed consent were invited to participate in the study. Patients with mentally unstable conditions, those with life threatening conditions and patients who were already in the hospital at the time of onset of symptoms of AMI were excluded from our study.

A structured questionnaire was used to collect data. Components of the questionnaire were taken from published studies [9,10,21,22]. The questionnaire was initially developed in English and then translated into Urdu, the national language of Pakistan. To ensure accuracy of the English to Urdu translation, the questionnaire was back translated from Urdu into English. The majority of the questions were close ended.

This analysis focused on identifying the level of knowledge about the modifiable risk factors for CVD [9]. Four aspects of knowledge of the modifiable risk factors for heart disease were assessed: a) fatty food consumption b) smoking c) obesity and d) lack of exercise [23]. Subjects were asked about the association of each risk factor, as well as direction of association, with heart disease [see additional file 1]. For each risk factor, if the subject correctly identified the association and the direction of the association of the risk factor with heart disease s/he would get a score of one otherwise zero for that component. Subjects scoring a total greater than or equal to three, out of a possible total of four were regarded as having a good level of knowledge of CVD risk factors. The questionnaire also incorporated additional questions designed to cross check validity of subject responses. Multiple logistic regression models were constructed for the primary outcome of having a good level of knowledge of CVD risk factors. We also analyzed each of these four modifiable risk factors separately, which enabled us to identify specific knowledge gaps in our population.

The independent variables of this study were age, gender, ethnicity (defined according to mother tongue) [24], marital status, level of education (years formal education was defined as person who had ever attended school), type of family system (nuclear family system was defined as a household consisting of two parents and their legal children; extended family system was defined as a household where multiple generations of family were living together), income, occupation, history of hypertension (self reported and subjects who were taking antihypertensive medication were defined as hypertensive), history of diabetes (self reported and subjects who were taking anti diabetic medication were defined as diabetic) and general health behavior including tobacco use, physical activity, number of visits to any health care facility in the previous year and knowledge of symptoms of a myocardial infarction (self reported).

Descriptive analyses were performed to assess the distribution of our data. Univariate logistic regression was run to determine the relationship of each independent variable with the outcome variables. All variables with a p-value < 0. 25 in the univariate analysis were included in multiple logistic regression model, and retained if they were significantly associated with the primary outcome in the final model (p < 0.05) [25]. Age and hypertension were included at all stages of the logistic regression analysis, because of their known importance. Confounding variables (change in the beta coefficient greater than 10%) were also retained in the model. [26]. Odds Ratios and 95% CI were reported to interpret our final model.

The study was approved by the Ethical Review Committee of the Aga Khan University and permission from the NICVD obtained. After explaining the purpose of the study to the participants, the informed consent form was read out clearly and verbal or written consent was requested. The questionnaire was administered by a trained research medical officer who read it out to the study subjects in Urdu. Questionnaire was read out to them by a trained research medical officer in Urdu. The participants had the right to refuse to answer any specific question or withdraw from the study at any time.

## Results

We interviewed 720 subjects. The response rate was 100%. Seventy eight percent of the subjects were male. The mean age (SD) was 54 (11.6) years (Table 1). Only 16% had completed more than 10 years of education. Around one third of married subjects were related to each other before marriage. Two thirds of the subjects lived in an extended family system. Around half of the subjects reported that they had never smoked. Only 42% of the subjects had a good level of knowledge of the risk factors of heart disease. Almost all subjects 693 (96%) were able to identify at least one risk factor. However, only 143 (20%) participants correctly identified the relationship of all four modifiable risk factors with heart disease (Table 2).

**Table 1:** Characteristics of the subjects with acute myocardial infarction in Karachi, Pakistan (n = 720)

**Characteristics**	**n (%)**
**Demographic and socio-economic Characteristics**	
Age	
Mean (SD)	54.1 (11.6)

Sex	
Male	562 (78.1)

Ethnicity	
Muhajir	524 (72.8)
Sindhi	61 (8.5)
Punjabi	44 (6.1)
Pushto	28 (3.9)
Balochi	16 (2.2)
Others ^§^	47 (6.5)

Marital status	
Single	28 (3.9)
Married	645 (89.6)
Separated	01 (0.1)
Widowed	46 (6.4)

Literacy Status	
*Can read & write a paragraph (3 line)*	
Yes	471 (65.4)
*Received formal (school) education*	
None (defined as Illiterate)	25 (3.5)
≤ 5 years	130 (18.0)
6–10 years	197 (27.4)
> 10 year	119 (16.5)

Parents related prior to marriage	
Yes	246 (34.2)
Type of relationship	
First cousins^+^	217 (30.1)
Second cousins *	29 (4.0)

Family history of CVD (first degree relative)	
Yes	265 (36.8)

Types of family system	
Nuclear	246 (34.2)
Extended	474 (65.8)

Income (PKRS)	
Median (IQR⊥)	4500 (4000)
≤ 3000	185 (25.7)
3001–5000	241 (33.5)
5001–8000	151 (21.0)
> 8000	101 (14.0)
Did not reveal	42 (5.8)

**General health behaviors**	
Tobacco use	
Never used	360 (50.0)
Current user	141 (19.6)
Ex-user	219 (30.4)
Exercise ^€^	
Yes	31 (4.3)
Number of visits for any health facility in past one year	
≤ 4	302 (41.9)
5 – 6	249 (34.6)
≥ 7	169 (23.5)

**Clinical Characteristics**	
Hypertension	
Yes	312 (43.3)
Diabetes	
Yes	197 (27.4)

§= include Rajistani, Bengali, Gujrati, Marhwi, Katchi, Sirayki or Hindi

+ = first cousins are the people, who have the same grandparents. (Children of aunts and uncles)

* = Second cousins are the people who do not have the same grandparents.

⊥ = IQR (Inter quartile range)

€ = Exercise (that lasted for at least 20 minutes with hard breathing and sweating)

**Table 2:** Knowledge of modifiable risk factors for CVD in patients admitted with acute myocardial infarction in Karachi, Pakistan (n = 720)

**Characteristics**	**n (%)**
Knowledge of modifiable risk factors	
Don't know any risk factor	27 (3.8)
Only one risk factor	89 (12.4)
A total of two risk factors	302 (41.9)
Good level of knowledge*	302 (41.9)

Knowledge of symptoms of heart attack	
Don't Know	586 (81.4)
List 1 symptom	93 (12.9)
List at least 2 symptoms	41 (5.7)

*defined as identifying three out of four modifiable risk factors (smoking, lack of exercise, obesity and consumption of fatty foods

**Figure 1:**
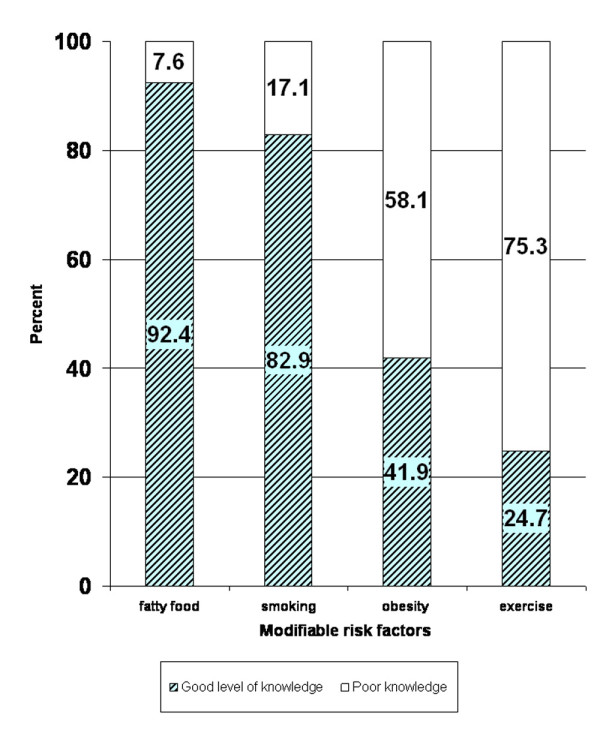
Percentage of knowledge of modifiable risk factor of heart disease among 720 subjects with acute myocardial infarction.

Out of 720 study subjects, 665 (92%) had good level of knowledge about the association of fatty food consumption with heart disease, 597 (83%) were able to correctly identify the association of smoking with heart disease, 302 (42%) were knowledgeable about the association of obesity with heart disease, and only 178 (25%) knew about the protective effect of exercise (figure 1).

Table 3 shows the crude, and adjusted odds ratios and 95% CI of our patient's knowledge of modifiable risk factor for heart disease. We found that subjects of Sindhi ethnicity, who had more year's formal of education, a higher level of exercise, a higher knowledge of symptoms of AMI and who lived in a nuclear family system, or did not use tobacco were more likely to have a good level of knowledge of modifiable risk factors of heart disease. These variables were adjusted for age, gender, profession, socioeconomic status, and history of hypertension because these were potential confounders.

**Table 3:** Crude and adjusted OR of knowledge of modifiable risk factors for heart diseases among subjects with first heart attack in a tertiary care hospital Karachi Pakistan. (n = 720)

Variable	Crude OR	95% CI	aOR*	95% CI
Age				

Older > 45 yrs	1.00		not significant	
Younger ≤45	1.41	(0.95, 2.09)		

Sex				
Female	1.00		not significant	
Male	2.81	(1.59, 4.96)		

Ethnicity				
Muhajir (Urdu)	1.00		1.00	
Sindhi	2.12	(1.81, 3.82)	3.03	(1.54, 5.95)
Punjabi	2.19	(1.12, 4.30)	1.90	(0.86, 4.19)
Pushtoo	0.57	(0.17, 1.91)	1.09	(0.29, 4.11)
Others	1.47	(0.79, 2.73)	1.33	(0.64, 2.77)

Years of school education				
Illiterate	1.00		1.00	
< 5 years	0.85	(0.45,1.58)	0.66	(0.33, 1.31)
6–10 years	1.73	(1.08, 2.79)	1.17	(0.67, 2.03)
> 10 years	3.24	(2.14, 5.85)	2.50	(1.30, 4.83)

Exercise				
No	1.00		1.00	
Yes	4.12	(1.98, 8.53)	2.76	(1.21, 6.28)

Type of family				
Extended	1.00		1.00	
Nuclear	3.14	(2.16, 4.58)	2.54	(1.65, 3.89)

Tobacco use				
Current user	1.00		1.00	
Ex user	0.46	(0.10, 2.26)	0.45	(0.08, 2.42)
Never used	2.28	(1.11, 4.69)	2.53	(1.15, 5.56)

Income				
> 8000	1.00			
5001–8000	0.69	(0.40, 1.21)	not significant	
3001–5000	0.39	(0.23, 0.66)		
≤ 3000	0.35	(0.20, 0.63)		
Didn't reveal	0.41	(0.17, 1.03)		

Hypertension				
No	1.00		not significant	
Yes	0.81	(0.56, 1.17)		

*Adjusted for confounding variable age, sex, profession, socioeconomic status (income) and history of hypertension

## Discussion

To the best of our knowledge, this is the first study of knowledge of risk factors for heart disease and its determinants in this high risk population of Pakistan. Knowledge of modifiable risk factors for heart disease, i.e. fatty food consumption, smoking, obesity, and exercise, was assessed among patients diagnosed with their first acute myocardial infarction at a tertiary care hospital. We found that subjects of Sindhi ethnicity, who had more year's formal of education, a higher level of exercise, who lived in a nuclear family system or who did not use tobacco were more likely to have a good level of knowledge of modifiable heart disease risk factors.

The present study also demonstrates that 96% of participants were able to recognize at least one risk factor for heart disease, but only 20% of the participants were fully aware of all four key modifiable risk factors of heart disease. Most of participants identified the association of fatty food consumption and smoking with heart disease (i.e. 92% and 83%). However, only minority could identify obesity or lack of exercise as risk factors for heart disease. These findings are similar to those of a recent study looking at cardiovascular knowledge in patient attendants as a surrogate for the general Pakistani population [18]. This pattern of knowledge may be due to relatively more aggressive state-funded advertising campaigns as well as educational programs discouraging the use of saturated fats and tobacco and a relative dearth of the same for obesity and exercise.

There was a strong, positive and independent association between the level of education and a good level of knowledge of modifiable risk factors of heart disease. This is consistent with previous studies [9-11,18,21,27-30]. Individuals who are better able to read are more able to understand health messages that are conveyed through print media and/or visual media. This finding identifies the need for targeting illiterate (and poorly educated) individuals in Pakistan with educational programs that are tailored to their level of understanding.

A significant majority of study participants (66%) were noted to be living in an extended family system. Interestingly, subjects who lived in an extended family system had less knowledge of risk factors for heart disease than those living in a nuclear families. One would have thought that those living in an extended family would have been more likely to be exposed to close relatives with heart disease, and thus to the medical advice that was offered, but the data do not support this. There are several possible explanations of this observation. First, the frequent and close contact of an extended family system may lead to exposure and reinforcement of traditional and potentially unhealthy health practices and beliefs [31]. Second, extended family living is frequently a necessity owing to the economic constrains of living independently. Thus persons living in extended families are more likely to be poor and less educated – a likely explanation of their lower level of knowledge. Third, people living in an extended family system may be more susceptible to depression and anxiety. [32] which may contribute towards less knowledge. To the best of our knowledge, the association between type of family system and level of knowledge of risk factors for heart disease has not been previously reported.

In this study, current and ex tobacco users were less knowledgeable about risk factors for heart disease than those who never used tobacco. This finding was consistent with other studies conducted in the US and Scotland [11,22]. One explanation is that tobacco users are not aware of the adverse consequences of their habit. On the other hand, it may be that tobacco users are simply less prepared to admit the health implications of what we commonly perceive as an unhealthy practice, at least in regard to their own health [22].

Earlier studies have shown a clear distinction in various health beliefs and health-seeking behavior among different ethnic groups [33,34]. In this study, Sindhi subjects showed higher levels of knowledge of the modifiable risk factors for heart disease than Urdu speaking people. However in the analysis of each modifiable risk factors separately, Sindhi participants had less knowledge of the relationship of fatty food consumption with heart disease compared to Urdu speaking. These ethnic differences in the knowledge base are not surprising since the various ethnic groups in Pakistan have significantly different cultural practices and beliefs [33]. However, these associations within these ethnic groups need to be further explored in future studies.

The American Heart Association has recently focused on physical inactivity as a major modifiable risk factor for heart disease [35]. In this study, 75% of the study subjects could not identify the relationship of exercise with heart disease. A study conducted in the US also reported that only 15% of the subjects recognized lack of exercise as a cause of heart disease [36]. In our study very few people (4%) exercised on a regular basis which is consistent with previous finding [37]. Our study showed that people who exercised regularly were more knowledgeable about the relationship of modifiable risk factors for heart disease, which is also consistent with Ford's study [11]. This finding highlights a potential area of emphasis for future educational programs.

A very worrying finding was that 81% of the study participants were not aware of any symptoms of heart attack and only 6% could identify two or more symptoms. This may increase the delay in seeking early medical care among AMI subjects, which would lead to a worse outcome. Therefore, we should also educate our population about the symptoms of heart attack along with risk factors of heart attack.

Our study has few limitations. First, Level of knowledge was assessed using a structured questionnaire. Subjects may have responded positively to all risk factors introduced, knowing that the study was about heart disease and risk factors, so we may have overestimated the total level of risk factor knowledge in this population. However, this cannot account for the striking difference in knowledge between certain groups, eg those classified by education or ethnic subgroup. Secondly, reliance of ability to recall risk factors for CVD introduces recall bias and may underestimate knowledge. Third, our questionnaire has not been validated; however, components of our questionnaire have been validated in other studies [9,10,21,22]. The content validity of questionnaire items was examined by the clinical experts and peer review. Fourth, the choice of a cumulative score of 3 as "good" knowledge is somewhat arbitrary; however we feel that this cut-off provides reasonable discrimination between those who are knowledgeable and those who are not. Finally, due to the cross sectional study design, one cannot determine the level knowledge status *prior *to the index event. It is likely that individual patients may have acquired some knowledge during their hospital stay as a direct consequence of being questioned about them during their admission. However, as our study subjects were recruited on the day following the AMI, we feel that a relatively limited degree of knowledge would have been accrued in the hospital and the knowledge assessed largely reflects their pre-event status.

## Conclusion

Our findings highlight a striking lack of knowledge of modifiable risk factors among individuals with a first heart attack. Only 42% of our subjects had a good level of knowledge about heart disease, and a mere of 20% were able to correctly identify all four risk factors of heart diseases in the Pakistani population. The results of this study have also helped to identify segments of the population who need to be targeted for educational interventions. These include tobacco users, people who have not completed high school education, people with a sedentary lifestyle, and those living in an extended family system and those of Urdu-speaking ethnic origin. The findings of our study suggest that aggressive and targeted education about the relationship of obesity and exercise with CVD in particular is needed.

Our study calls for efforts such as targeted public health education to increase the level of knowledge of risk factors of heart disease. Education can be provided to the public through the media (including both electronic and print) and workshops. These programmes must be sensitive to the attitudes, perceptions and capabilities of targeted individuals. Physicians must also ensure they impart education to their patients, as patients usually rely on doctors for first hand information. Further epidemiological studies, especially longitudinal studies and population based, are needed in Pakistan to assess the level of knowledge regarding modifiable behavioral risk factors for heart disease.

## Competing interests

The author(s) declare that they have no competing interests.

## Authors' contributions

MS did the study under the supervision of TJ and performed the statistical analysis, and drafted the manuscript. JH supervised the statistical analysis. FH, IR&; AF provided the clinical knowledge of AMI. TJ, JH and NC contributed to review, and to the revision of the report. All authors read and approved the final manuscript

## Pre-publication history

The pre-publication history for this paper can be accessed here:



## Supplementary Material

Additional File 1 Knowledge about the modifiable risk factors of heart disease. Its one of the part of our questionnaire in which we asked questions about the knowledge of modifiable risk factors of heart disease from our study patricians. Click here for file
