# Supplementary material for: Knowledge of modifiable risk factors of heart disease among patients with acute myocardial infarction in Karachi, Pakistan: a cross sectional study
Source: BMC Cardiovasc Disord. 2006 Apr 27;6:18. doi: 10.1186/1471-2261-6-18 (PMC1464150; doi:10.1186/1471-2261-6-18)
Supplement: Additional File 1 — Knowledge about the modifiable risk factors of heart disease. Its one of the part of our questionnaire in which we asked questions about the knowledge of modifiable risk factors of heart disease from our study patricians. [file 1471-2261-6-18-S1.doc]

**Appendix1**

**Knowledge about the modifiable risk factors of heart disease**

| **Q no** | **Questions** | **Code** | **Skip** |
| --- | --- | --- | --- |
|  | **Fatty foods consumption**  Is there any relationship between fatty food consumption and heart attack? | 1. **Yes** 2. **No** 3. **Don’t Know** | **If 2 or 3 skip to Q3** |
|  | How does consumption of fatty food affect the heart attack? | 1. **Increases** 2. **Decrease** 3. **No effect** 4. **Don’t Know** |  |
|  | **Vegetables**  Is there any relationship between diet rich in vegetables on heart attack? | 1. **Yes** 2. **No** 3. **Don’t Know** | **If 2 or 3 skip to Q5** |
|  | What is the affect of diet rich in vegetables on heart attack? | 1. **Increase** 2. **Decrease** 3. **No effect** 4. **Don’t Know** |  |
|  | **Fruits**  Is there any relationship between diet rich in fruit on heart attack? | 1. **Yes** 2. **No** 3. **Don’t Know** | **If 2 or 3 skip to Q8** |
|  | What is the affect of diet rich in fruit on heart attack? | 1. **Increase** 2. **Decrease** 3. **No effect** 4. **Don’t Know** |  |
|  | In your opinion which product ghee or oil is better? | 1. **Oil is better** 2. **Ghee is better** 3. **No difference** 4. **Don’t Know** |  |
|  | **Smoking**  In your opinion is there any relationship between smoking and heat attack? | 1. **Yes** 2. **No** 3. **Don’t Know** | **If 2 or 3 skip to Q10** |
|  | In your opinion how does smoking affect the heart attack? | 1. **Increases** 2. **Decrease** 3. **No effect** 4. **Don’t Know** |  |
|  | **Exercise**  In your opinion is there any relationship between exercises and heart attack? | 1. **Yes** 2. **No** 3. **Don’t Know** | **If 2 or 3 skip to** **Q12** |
|  | In your opinion how does exercise affect heart attack? | 1. **Increase** 2. **Decrease** 3. **No effect** 4. **Don’t Know** |  |
|  | **Obesity**  Is there any relationship between obesity and heart attack? | 1. **Yes** 2. **No** 3. **Don’t Know** | **If 2 or 3skip to Q14** |
|  | In your opinion how does obesity affect heart attack? | 1. **Increases** 2. **Decreases** 3. **Don’t Know** |  |
